# Role of apalutamide in the treatment landscape for patients with advanced prostate cancer: an expert opinion statement of European clinical practice

**DOI:** 10.1007/s11845-023-03303-y

**Published:** 2023-03-22

**Authors:** Martin Bögemann, Gaetano Facchini, Thomas Bauernhofer, Richard Cathomas, Evanguelos Xylinas, Bertrand Tombal

**Affiliations:** 1 Department of Urology, Münster University Medical Centre, Münster, Germany; 2 Oncology Complex Unit, “S. M. Delle Grazie” Hospital, ASL Napoli 2 Nord, Pozzuoli, Italy; 3 Division of Clinical Oncology, Department of Internal Medicine, Medical University of Graz, Graz, Austria; 4 Division of Oncology/Hematology, Kantonsspital Graubünden, Chur, Switzerland; 5 Department of Urology, Bichat-Claude Bernard Hospital, Université Paris Cité, Paris, France; 6 Division of Urology, Cliniques Universitaires St-Luc, Université Catholique de Louvain, Brussels, Belgium

**Keywords:** Apalutamide, Metastatic hormone-sensitive prostate cancer, Non-metastatic castration-resistant prostate cancer, Prostate cancer

## Abstract

**Background:**

Patients with advanced prostate cancer have a poor prognosis, and well-tolerated new treatment strategies are required to improve survival outcomes. Apalutamide is a novel androgen signalling inhibitor developed to be used in combination with continuous androgen deprivation therapy (ADT) for the treatment of patients with advanced prostate cancer. Based on evidence from two phase 3 pivotal clinical trials in non-metastatic castration-resistant (nmCRPC; SPARTAN) and metastatic hormone-sensitive prostate cancer (mHSPC; TITAN), ADT plus apalutamide significantly extends overall survival compared with the standard of care.

**Aims:**

To provide practical recommendations to guide optimal use in the real-world setting as the use of apalutamide in clinical practice increases.

**Methods:**

Expert opinion from a group of European physicians is presented here to educate on the use of apalutamide in combination with ADT in patients with mHSPC and patients with nmCRPC who are at risk of developing metastatic disease, focusing on practical considerations such as patient selection, monitoring, and management of side effects.

**Results:**

In clinical practice, apalutamide in combination with ADT can be used in a broad patient population including patients with high and low volume/risk mHSPC, patients with de novo metastatic disease or metastases following treatment for localised disease, as well as older patients. Apalutamide in combination with ADT is well tolerated, with manageable side effects which do not impact health-related quality of life compared to ADT alone.

**Conclusions:**

Real-world experience with apalutamide supports the efficacy and safety findings reported by the SPARTAN and TITAN clinical trials.

## Overview of the role of apalutamide in the management of advanced prostate cancer

### Prostate cancer disease burden

Worldwide, prostate cancer is the second most common cancer diagnosed in men; it is responsible for approximately 7% of all male cancers and is the fifth most common cause of cancer-related death among men [1]. In more than half of all the countries of the world, it is the most frequently diagnosed cancer in men, and approximately 1.4 million new cases of prostate cancer are diagnosed annually [1]. Incidence rates vary from 6.3 to 83.4 cases per 100,000 men with the highest rates observed in Northern and Western Europe, North America, Australia and New Zealand, and Southern Africa [1]. The prognosis of prostate cancer is highly variable and dependent on the tumour grade and stage at initial diagnosis; patients diagnosed early with localised disease confined to the prostate can have a life expectancy of 99% over 10 years, whereas patients diagnosed with late stage, advanced disease with distant metastases have poor survival of only approximately 30% at 5 years [2]. Due to its prevalence, and the increase in diagnosis of early-stage tumours through screening, many patients live with prostate cancer for years or even decades, and it is estimated that at least 10 million men are living with prostate cancer of whom 700,000 have metastatic disease [3]. Metastatic prostate cancer is responsible for > 400,000 cancer-related deaths annually, and a similar number of patients live with treatment-related morbidity 10 years after diagnosis [3, 4]. Androgen deprivation therapy (ADT) is the backbone systemic treatment for advanced prostate cancer, although patients with aggressive disease will eventually relapse and experience disease progression. Effective, well-tolerated new treatments that delay the development of metastases and improve survival outcomes for patients with advanced disease are therefore needed to reduce the global burden of disease caused by prostate cancer.

## Current treatment approaches and unmet needs for patients with advanced prostate cancer

### Metastatic hormone-sensitive prostate cancer

Patients who present with metastatic prostate cancer are a heterogeneous population. Metastases, usually in the lymph nodes, bone, or lung and liver, can be present at the initial diagnosis of prostate cancer (de novo or synchronous metastases) or may develop after treatment for localised disease (metachronous metastases). The exact proportion of each of these types of disease stages is not known and varies by country or clinical practice setting. In addition, metastatic hormone-sensitive prostate cancer (mHSPC) can be classified as either high/low volume or high/low risk [5, 6]. Using definitions from the CHAARTED trial, high-volume mHSPC disease is defined as the presence of visceral metastases and/or ≥ 4 bone lesions with ≥ 1 outside of the spine or pelvis [5]. Alternatively, mHSPC can be defined as either high or low risk using the definitions used in the LATITUDE trial which considered high-risk disease to be the presence of at least two of the following: Gleason Score ≥ 8, ≥ 3 bone metastases (independent of location), and/or visceral metastases [7]. Importantly, high-volume (or high-risk) disease and de novo metastatic diagnosis have been shown to be independently associated with a poor prognosis in mHSPC [8], and there is a significant clinical need for novel treatment regimens to improve outcomes in these patients.

For patients with mHSPC, intensification of ADT with different agents including docetaxel [5, 9], abiraterone acetate plus prednisone (AAP) [7, 10, 11], enzalutamide [12, 13], or apalutamide [14, 15] has been shown to improve overall survival (OS) and is currently recommended for mHSPC patients (Table 1; [16]).

**Table 1:** 2022 EAU recommendations for nmCRPC and mHSPC

Indication	Recommendation	Trial
nmCRPC	ADT + apalutamide ADT + enzalutamide ADT + darolutamide	SPARTAN [21] PROSPER [23] ARAMIS [25]
mHSPC	ADT + apalutamide ADT + docetaxel	TITAN [15] STAMPEDE [9] CHAARTED [5]
	ADT + abiraterone acetate + prednisone	LATITUDE [7] STAMPEDE [10]
	ADT + enzalutamide ± docetaxel	ENZAMET [13]
	ADT + enzalutamide	ARCHES [12]

*ADT* antigen deprivation therapy, *mHSPC* metastatic hormone-sensitive prostate cancer, *nmCRPC* non-metastatic castration-resistant prostate cancer

The treatment landscape for mHSPC continues to evolve rapidly. Triplet combinations of ADT plus docetaxel and either abiraterone acetate plus prednisone [17] or darolutamide [18] demonstrated superior OS when compared to ADT plus docetaxel alone. This triplet approach can be considered for patients with de novo high-volume/risk disease who are fit to receive chemotherapy treatment [19]. At the time of this article, triplet combinations were not yet evaluated by the EAU guidelines committee [16] for the treatment of patients with mHSPC. If these two trials are however suggesting that ADT plus docetaxel alone should not be considered a standard of care, they do not provide clarity on which patients needs docetaxel when ADT plus one of the available androgen receptor-targeted agents is the chosen treatment.

### Non-metastatic castration-resistant prostate cancer

Patients treated with ADT for biochemical relapse who progress with three consecutive rises in prostate-specific antigen (PSA) levels, a PSA of > 2 ng/mL, and a castrate serum testosterone level (< 50 ng/dL or 1.7 nmol/L) are considered by definition to have castration-resistant prostate cancer (CRPC) by EAU guidelines [16]. In the absence of detectable metastases on conventional imaging, these patients are classified as having non-metastatic CRPC (nmCRPC). Patients with nmCRPC are considered to have high-risk disease when their PSA doubling time is ≤ 10 months, as this has been associated with a higher risk of metastatic progression and death [20]. Due to the fact that high-risk nmCRPC is an asymptomatic disease, treatment aims to delay development of metastases and increase OS, while maintaining health-related quality of life (HRQoL). Delaying or preventing the progression to metastatic disease and prolonging OS are an important goal of management in patients with high-risk nmCRPC, along with preserving QoL. The development of effective treatment options for these patients has long been an unmet need. Until recently, treatment options for patients with high-risk nmCRPC were ADT alone until the development of metastatic disease. However, several key clinical trials (SPARTAN, PROSPER, ARAMIS) have demonstrated that an early introduction of apalutamide, enzalutamide, or darolutamide in high-risk nmCRPC patients significantly extends metastasis-free survival (MFS) and OS [18, 21–25]. Current EAU guidelines recommend that patients with high-risk nmCRPC (i.e. PSA doubling time ≤ 10 months) should receive treatment with ADT plus either apalutamide, enzalutamide, or darolutamide to delay metastasis and prolong life (Table 1; [16]).

## Apalutamide key clinical data

Apalutamide is a novel androgen signalling inhibitor developed for the treatment of advanced prostate cancer. Two key registrational phase 3 clinical trials provided clinical evidence for the efficacy and safety of apalutamide in combination with ADT: SPARTAN in patients with high-risk nmCRPC [21, 26] and TITAN in patients with mHSPC [14, 15].

### High-risk nmCRPC

In the SPARTAN trial, 1207 patients with high-risk nmCRPC diagnosed using conventional imaging were randomised in a 2:1 ratio to receive apalutamide 240 mg/day plus ADT or matched placebo plus ADT [21, 26]. Patient demographics were well balanced between the two treatment groups; approximately 16% of patients in the apalutamide and placebo groups had malignant pelvic lymph nodes that measured less than 2 cm in the short axis (classified as N1), the median patient age in both treatment groups was 74 years, and the median PSA doubling time was < 5 months. In patients treated with apalutamide plus ADT, the risk of metastasis or death was 72% lower than for patients on ADT alone (hazard ratio [HR] for metastasis or death 0.28, 95% confidence interval [CI] 0.23–0.35; *p* < 0.001), and the median MFS was more than 2 years longer with apalutamide plus ADT (median MFS 40.5 vs 16.2 months, respectively). Based on the data from the SPARTAN trial, apalutamide became the first US Food and Drug Administration (FDA) and European Medicines Agency (EMA) approved treatment for high-risk nmCRPC. In SPARTAN, a total of 19% of patients crossed over to apalutamide after the trial was unblinded when the primary endpoint of MFS was met. In the final OS analysis, the median OS was significantly longer with apalutamide versus ADT alone (73.9 vs 59.9 months), and apalutamide plus ADT was associated with a 22% reduction in the risk of death compared standard ADT only (HR 0.78, 95% CI 0.64–0.96; *p* = 0.016) despite the crossover [26]. Compared with placebo, there was a higher incidence of skin rash (24.8/14.3% vs 5.5/0.3%), hypothyroidism (8.0/0% vs 2.0/0%), bone fracture (11.7/2.7% vs 6.5/0.8%), fatigue (30.0/0.9% vs 21.1/0.3%), and falls (15.6/1.7% vs 9.0/0.8%) with apalutamide plus ADT for all grades and for Grade 3/4 adverse events (AEs), respectively [21].

### mHSPC

The double-blind, randomised phase 3 TITAN trial evaluated the efficacy and safety of apalutamide in combination with standard ADT in a broad population of patients with low- and high-volume/risk mHSPC, the majority of whom had newly diagnosed metastatic disease, but the population also included patients with metachronous mHSPC [14, 15]. Overall, 1052 patients with mHSPC were randomised in a 1:1 ratio to treatment with apalutamide 240 mg once daily or matched placebo in addition to standard ADT. Patients that did not experience disease progression in the placebo arm were permitted to cross over to apalutamide after the trial was unblinded; 39.5% of patients crossed over to apalutamide after unblinding. Patient demographics were well balanced between apalutamide and placebo cohorts [14]. Median age was 69 years of age in the apalutamide arm, and a total of 38% had low-volume and 62% high-volume disease. Previous treatment with docetaxel had been received by 11% of patients, and 18% had received prior therapy for localised prostate cancer and therefore presented with metachronous disease. All patients had bone metastases by conventional imaging at study entry; the proportion of patients with bone-only disease was 55% in the apalutamide arm, and the proportion of patients with visceral and bone metastases was 11%.

At the first interim analysis of TITAN data, with a median follow-up of 22.7 months, apalutamide in combination with ADT significantly improved OS (HR 0.67, 95% CI 0.51 to 0.89; *p* = 0.005). The final survival analysis from TITAN demonstrated that the use of apalutamide was associated with significant improvement in OS and delayed disease progression despite 39.5% of patients crossed over from placebo to apalutamide once the study was unblinded [15]. The risk of death was reduced by 35% (median OS not reached vs 52.2 months; HR 0.65, 95% CI 0.53–0.79; *p* < 0.0001) and 48% after adjusting for crossover of patients from the placebo arm (median OS not reached vs 39.8 months; HR 0.52, 95% CI 0.42–0.64; *p* < 0.0001) [15]. Apalutamide plus ADT also delayed the median time to second progression-free survival (PFS2) compared to placebo plus ADT (not reached vs 44 months; HR 0.62, 95% CI 0.51–0.75; *p* < 0.0001), and the time to castration resistance (not reached vs 11.4 months; HR 0.34, 95% CI 0.29–0.41; *p* < 0.0001).

Notably, the benefit of apalutamide plus ADT on OS was seen in patients with high-volume (not reached vs 14.9 months; HR 0.53, 95% CI 0.49–0.67) and low-volume (not reached vs 30.5 months; HR 0.36, 95% CI 0.22–0.57) disease. HRQoL was maintained throughout, up to 4 years of follow-up in patients with mHSPC [27]. Rapid declines of > 90% in PSA levels (PSA90) were observed in 14% of patients treated with apalutamide plus ADT in TITAN, and patients who achieved PSA90 or a PSA nadir of ≤ 0.2 ng/mL had a reduced risk of radiographic progression and extended MFS [28]. A total of 15% of patients achieved a PSA decline of > 50% (PSA50 response), and median time to PSA50 was 1 month [28]. Deep and rapid PSA declines in patients treated with apalutamide have been associated with prolonged time to deterioration in HRQoL in the TITAN and SPARTAN trials [29]. In the TITAN and SPARTAN studies, deep and rapid PSA responses with apalutamide were associated with prolonged time to deterioration in HRQoL, Functional Assessment of Cancer Therapy – Prostate (FACT-P) physical wellbeing, Brief Pain Inventory-Short Form (BPI-SF) worst pain intensity, and Brief Fatigue Inventory (BFI) worst fatigue intensity in patients with advanced prostate cancer [29]. The overall incidence of treatment-emergent AEs was similar between the apalutamide and placebo groups in TITAN, but rate of AEs of interest was increased with the addition of apalutamide skin rash (24.4/2.9% vs 8.3/0.6%), bone fracture (6.1/1.5% vs 1.3/0.8%), falls (4.6/0.7% vs 6.8/0.6%), ischemic heart disease (5.9/3.1% vs 2.1/0.8%), ischemic cerebrovascular disorder (2.5/1.6% vs 1.5/0.2%), and seizure (0.6/0.2% vs 0.4/0%) for all grades and for Grade 3/4 AEs, respectively [15].

## Expert opinion: role of apalutamide in clinical practice

Apalutamide is now an integral part of the clinical armamentarium for treating advanced prostate cancer and has been approved for use in combination with ADT in patients with high-risk nmCRPC and those with mHSPC. Real-world clinical experience needs to be added to clinical trial data to guide clinicians using apalutamide to optimally treat their patients with prostate cancer. The second part of this review is therefore based upon the expert opinion of the authors, a group of European urologists and medical oncologists, who summarised their clinical experience with apalutamide in the treatment of prostate cancer.

## Which patients are suitable for treatment with apalutamide?

Apalutamide is an oral treatment, administered as four 60-mg tablets given at the same time (total daily dose 240 mg), with or without food, and is prescribed in addition to standard continuous ADT. In current EAU guidelines, apalutamide plus continuous ADT is recommended as a first-line treatment option for men with mHSPC [16, 30]. Importantly, apalutamide was evaluated in a population of mHSPC “all comers”, meaning that all subpopulations within mHSPC were included. That is, apalutamide has demonstrated efficacy including mHSPC patients who have synchronous or metachronous disease that has metastasised following initial treatment for a localised tumour [31] and including patients with both high- and low-volume as well as both high- and low-risk disease. In patients with mHSPC treatment with apalutamide plus ADT should be early, ideally within 1 month of diagnosis of metastatic disease as early treatment intensification with apalutamide has been shown to be key to optimising outcomes. In the TITAN trial, PFS2 was longer in patients treated with apalutamide in combination with ADT, which was also noted in patients who crossed over from the placebo arm to the apalutamide arm, which supports the early use of apalutamide in mHSPC [14, 15]. In contrast to treatment intensifications with docetaxel and abiraterone acetate plus prednisone, that is mainly beneficial in high-volume disease, and with abiraterone that is licenced only in high-risk disease, the disease’s volume of metastatic disease does not restrict treatment selection for apalutamide. Indeed, TITAN shows that all patients, irrespective of the volume, risk status, or the timing of the metastases, benefit from early addition of apalutamide.

Apalutamide should also be considered for the treatment of men with high-risk nmCRPC since apalutamide plus ADT does delay the time to metastasis and improves OS in these patients [21, 22]. As described previously, high-risk nmCRPC relates to patients on continuous ADT who have developed castration resistance with a rapidly rising PSA level defined as a PSA-doubling time of ≤ 10 months.

### What other considerations are important when prescribing apalutamide for a patient?

It is necessary to look at the patient’s overall physical condition and assess all potential patients for their level of frailty, existing cognitive impairment, and overall life expectancy. Apalutamide is effective in older as well as younger patients with high-risk nmCRPC and mHSPC. Post hoc analyses of data from SPARTAN and TITAN trials have shown that apalutamide improves radiographic PFS (rPFS) and OS in patients ≥ 65 and 65–79 years of age [32]. Although rates of AEs, particularly rash, were increased in older patients, HRQoL was maintained in the population of older patients, and they did not report increased bother from treatment side effects [32]. However, patients who are very frail with limited life expectancy or with significant comorbidities may not be suitable for treatment with any regimen beyond standard ADT. This is an important clinical decision that must be taken on an individual basis following thorough evaluation of patient history, physical condition, and patient treatment preferences.

When prescribing apalutamide, comorbidities of relevance include dementia, severe organ insufficiency (i.e. heart, lung) leading to a life expectancy < 12 months and patients with an Eastern Cooperative Oncology Group (ECOG) performance status > 2. However, in the real-world setting, it is feasible and may be clinically reasonable to treat patients with cardiovascular disease, moderate renal insufficiency, and moderate liver impairment—this decision should be based upon individual patient history and their ability to tolerate the treatment. Cardiovascular contraindications include clinically significant cardiovascular disease within the past 6 months including myocardial infarction, severe/unstable angina, symptomatic congestive heart failure, or thromboembolic events [33]. Considerations for patients with renal impairment include an estimated glomerular filtration rate < 30 mL/min; and with hepatic impairment include elevated bilirubin or liver transaminase levels.

Apalutamide is a potent enzyme inducer cytochrome P450 (CYP)2C8 and CYP3A4 and as such may affect the efficacy of a number of other medications that may be prescribed concomitantly. This includes a number of drugs used to treat or prevent cardiovascular and thromboembolic disease (e.g. simvastatin, quinidine, amiodarone, gemfibrozil, clopidogrel, dabigatran etexilate, warfarin), blood pressure–lowering drugs (e.g. felodipine), anti-anxiety medications (e.g. midazolam, diazepam), certain antibiotics (e.g. clarithromycin, moxifloxacin), and antiviral drugs (e.g. ritonavir). It is therefore important to carry out a full review of patient’s current medications before prescribing apalutamide, particularly as many patients with advanced prostate cancer are older and may be taking medications for several comorbid conditions.

## The role of imaging in treatment decisions

Imaging is important to guide apalutamide treatment; however, the imaging method used can affect staging and varies by country. Computed tomography (CT) and bone scans are the traditional standard methods of imaging, but the availability of prostate-specific membrane antigen-guided positron emission tomography (PSMA-PET) scanning is growing, and this method is used in several European countries to assess prostate cancer patients because PSMA-PET can detect metastases earlier and at a smaller size. Current EAU guidelines acknowledge that PSMA-PET/CT is more accurate for staging prostate cancer than CT and bone scans, but currently there are no outcome data to guide/inform clinicians on disease management in relation to PSMA-PET/CT findings [16]. The heterogeneity of daily clinical practice in European cancer treatment centres and in different countries does not allow any conclusions to be drawn on optimal imaging strategy. Many patients with mHSPC, who are classified as having low-volume disease using conventional imaging with CT and bone scan, may be stage migrated if PSMA-PET imaging was used; and the same issue may also affect high-risk nmCRPC, when PSMA-PET is used [34]. Data from the pivotal SPARTAN and TITAN clinical trials were produced based on conventional imaging leading to the question of whether PSMA-PET scans are truly needed in a situation when high-risk nmCRPC is diagnosed using conventional imaging. Neither apalutamide nor darolutamide are approved for the treatment of mCRPC; therefore, increased use of PSMA-PET scans might lead to an earlier diagnosis of mCRPC in most patients, thereby missing an opportunity to use apalutamide or darolutamide in nmCRPC and leaving these patients with fewer treatment choices to fight their disease. The consensus of this group of authors was that disease staging should be carried out using conventional CT and bone scans, but it is important to note that clinical practice is evolving faster than clinical trial protocols with respect to next-generation imaging, and it is important to carry out clinical studies that use PSMA-PET imaging to assess disease stage at baseline and at follow-up assessments.

## Real-world clinical experience with apalutamide

PSA levels are an important clinical marker for response to treatment in prostate cancer. In the apalutamide pivotal clinical trials, rapid and deep declines in PSA levels (> 90%) have been reported in patients with high-risk nmCRPC and in those with mHSPC [28], and they are correlated with improved rPFS and MFS, improved OS [15], and improved HRQoL in patient-reported outcomes [29]. These findings are supported by the clinical experience of the authors with apalutamide, as well as emerging evidence from clinical practice. Published real-world evidence is very limited, but a recent study analysed data from patients with mHSPC treated with ADT plus either apalutamide or enzalutamide in 69 urology clinics in the USA. PSA responses were observed with both treatment regimens, but initiation of apalutamide in addition to ADT was associated with significantly faster and deeper PSA declines than enzalutamide [35].

Regular patient monitoring is important during treatment with apalutamide plus ADT. While there is still some debate on the frequency of PSA monitoring, if feasible, patients should be seen monthly for the first 3 months and then on a 3-monthly basis thereafter to monitor effectiveness of treatment and to ensure patient compliance. Patients on long-term treatment regimens visit the oncology clinic every 3 months for their injection of ADT, and this can be linked to their PSA monitoring. As described previously, PSA levels are key indicators of treatment efficacy in prostate cancer, which is particularly important given the high cost of treatment. Although not mandated in the prescribing information for apalutamide, laboratory tests should be carried out before starting treatment and at regular intervals to check liver, renal, and thyroid function.

Additionally, imaging should be performed initially after 3 to 6 months and then every 6 months using CT and bone scans to confirm the patient’s disease stage and assess for metastases. In line with the HRQoL evidence from the SPARTAN and TITAN trials, clinical experience in Europe confirms that patients are generally very well able to tolerate the ADT plus apalutamide treatment regimen, maintaining a favourable QoL. The recommendations of the authors for monitoring patients being treated with apalutamide in combination with ADT are summarised in Table 2.

**Table 2:** Expert panel recommendations for clinical assessment and management of patients receiving apalutamide in combination with ADT

	Clinical assessment/tests	Notes
Before initiating treatment	Clinical history—review for cardiovascular events and concomitant medications Assessment of comorbidities/patient frailty Assess fracture/fall risk Consider baseline thyroid function test	If patient has a history of cardiovascular disease or cardiovascular events in past 6 months, refer to cardiologist before starting treatment Patients with a history of osteopenia/previous fractures/frailty need careful management (physiotherapy, vitamin D and/or calcium/antiresorptive agents) due to potential impact of apalutamide plus ADT on BMD
Each clinic visit (monthly initially, then every 3 months)	Check PSA level Assessment of liver, kidney, and thyroid function Review of any patient-reported side effects (fatigue, skin rash)	
Every 6 months	CT imaging and bone scan OR PSMA-PET	

*ADT* antigen deprivation therapy, *BMD* bone mineral density, *CT* computed tomography, *PSA* prostate-specific antigen, *PSMA-PET* prostate-specific membrane antigen-positron emission tomography

## Management of AEs with apalutamide

The most common AEs associated with apalutamide in clinical trials were skin rash (29.2% of any grade), fatigue (20.4%), hypertension (19.5%), hot flush (23.1%), arthralgia (19.7%), and increased fracture risk (10.3%) [15].

### Skin rash

A mild macular or maculopapular skin rash is a common side effect associated with apalutamide treatment that usually occurs early in the treatment course [22, 36]. In the clinical trials of apalutamide, the median time until the first manifestation of skin rash was 83 days, and in 78% of patients, the rash disappeared after a median of 78 days [33]. Grade 1 skin rash (< 10% body surface area [BSA]) associated with apalutamide can be successfully controlled through careful monitoring and dose adaption. The use of antihistamine tablets or topical corticosteroids is also recommended. The notion that apalutamide-associated skin rash is being attributed to off-target pharmacological reactions [37, 38] suggests that monitoring and dose adaption interventions are crucial for rash management. For Grade 2 skin rash (10–30% BSA), patients should interrupt apalutamide treatment for a few days until the rash is resolved using antihistaminics, topical corticosteroids, or a short course of oral corticosteroids. When apalutamide treatment is resumed after a Grade 2 skin rash, a dose reduction should be considered, restarting treatment at either 180 or 120 mg of previous dose. If the rash does not recur, the dose can be re-escalated to 240 mg. In the clinical experience of the authors, Grade 2 skin rash does not recur frequently and is rarely seen after 6 months of treatment. Grade 3 skin rash (> 30% BSA) on apalutamide should always be managed by interrupting treatment and controlled using a topical corticosteroid, oral antihistaminics, antihistamine, and a systemic corticosteroid. Patients who are receiving apalutamide should be seen in the clinic regularly and should be educated about the likelihood of skin rash before starting and advised to see their doctor without delay if it occurs. Clinicians may consider counselling patients that the onset of skin rash typically occurs within the first 3 months of treatment; if a rash has not manifested after 6 months of treatment, then it is highly unlikely to develop.

### Hypothyroidism

As a potent enzyme inducer, apalutamide may induce uridine 5′-diphospho-glucuronosyltransferase (UGT). Thyroid hormone (T4) is metabolised by UGT, and consequently induction of UGT may promote faster metabolism and clearance of T4, with subsequent increases in the level of thyroid-stimulating hormone (TSH) [36]. In clinical practice, it is advised that patients’ thyroid function should be checked before start of treatment and at each clinic visit to detect hypothyroidism. In case of occurrence of hypothyroidism, hormonal substitution is advised.

### Cardiovascular events

Cardiovascular events are not common with apalutamide, but every patient requires a thorough clinical history and evaluation of comorbidities which should include a history of cardiovascular events 6 months prior to starting apalutamide. If a patient is known to have active cardiovascular disease, a cardiology consultation is needed before prescribing apalutamide. In such cases, treatment can be initiated with ADT alone, and apalutamide can be added once the patient has approval by the cardiologist.

### Fractures

Prostate cancer patients, particularly those with advanced disease, are typically older and as such are at higher risk of falls and fractures. By itself, ADT is associated with decreased bone mineral density and increased fracture risk [20], and apalutamide may further increase this risk [21]. In the pivotal clinical trials, when apalutamide was added to ADT, the prevalence of bone fractures was 18% in SPARTAN [26] and 10.3% in TITAN [15]. All patients who are on ADT, and even more for those considered for treatment intensification with apalutamide, should be carefully evaluated for fracture risk using dual energy X-ray absorptiometry (DEXA) measurement and prediction tools such as the FRAX (Fracture Risk Assessment Tool, Centre for Metabolic Bone Diseases, University of Sheffield, UK) score and should receive appropriate interventions to mitigate their risk such as physiotherapy and vitamin D/calcium supplementation and/or bone-targeting agents such as denosumab, zoledronic acid, or alendronate as preventive measures for the development at the osteoporosis prevention dose as well [16].

### Fatigue

In clinical practice, increased fatigue is reported by some patients during treatment with apalutamide plus ADT, but the level of fatigue related to apalutamide is difficult to assess separately from the impact of advanced prostate cancer per se and the effect of ADT—both of which are known to increase fatigue [2]. In the TITAN trial, the addition of apalutamide to ADT did not worsen fatigue to a clinically relevant degree in patients with mHSPC; energy levels were maintained at each treatment cycle in 78% of patients [24]. In the experience of the expert panel to date, the dose of apalutamide rarely needs to be reduced due to fatigue, although fatigue can be an issue for elderly and frail patients. Practical recommendations to manage fatigue associated with treatment include taking apalutamide tablets before bedtime and keeping levels of daytime physical activity high. Dose reduction can also be helpful for some elderly or frail patients; the authors suggest considering a 60 mg reduction in dose tapering to a 120 mg dose reduction if needed.

## Quality of life

Most asymptomatic and high-risk nmCRPC patients are very focussed on their PSA levels, and their QoL is strongly related to having declining/low PSA at follow-up visits. They are often willing to live with other, relatively mild, adverse effects of apalutamide treatment, such as skin rash, as a “trade-off” for steep declines in PSA. Overall, very little adverse effect management or support is needed with apalutamide for both mHSPC and high-risk nmCRPC patients, which is considered a very well-tolerated treatment, and patients usually discuss any QoL issues or adverse effects of treatment at their regular clinic visit (either monthly initially or every 3 months during long-term treatment). Although not formally reported, the clinical experiences of the authors show that QoL with apalutamide in the real-world setting seems to be comparable to that reported in the TITAN and SPARTAN clinical trials [27].

## Conclusions

Apalutamide, used in combination with ADT, is an important addition to the rapidly evolving treatment landscape in prostate cancer and can improve survival for patients with mHSPC and those with high-risk nmCRPC. Real-world experience with apalutamide supports the efficacy and safety findings reported by the TITAN and SPARTAN clinical trials; in clinical practice, apalutamide in combination with ADT can be used in a broad patient population including patients with high- and low-volume/risk mHSPC, patients with de novo metastatic disease or metastases following treatment for localised disease, as well as older patients. Importantly, intensification of ADT with apalutamide can significantly delay the development of metastases, as well as prolonging OS in patients with high-risk nmCRPC who, until recently, have had limited treatment options. Physicians prescribing apalutamide should conduct a thorough assessment of the patient’s history and current medications and monitor patients regularly during treatment to assess effectiveness and manage any side effects, particularly skin rash during the first 6 months of treatment. Apalutamide in combination with ADT is a generally well-tolerated regimen, with manageable side effects that do not usually increase the overall treatment burden for patients or further impact their disease-related QoL.
